# Two new species of *Beraba* Martins, 1997 and new geographical records of Eburiini (Coleoptera, Cerambycidae)

**DOI:** 10.3897/zookeys.827.31469

**Published:** 2019-03-05

**Authors:** Kimberly García, Juan Pablo Botero, Neis José artínez

**Affiliations:** 1 Semillero de investigación Artrópodos NEOPTERA del Caribe Colombiano. Programa de Biología, Facultad de Ciencias Básicas, Universidad del Atlántico, Carrera 30 # 8–49 Puerto Colombia, Atlántico, Colombia Universidad del Atlántico Atlántico Colombia; 2 Laboratório de Coleoptera, Museo de Zoologia, Universidade de São Paulo, Av. Nazaré, 481 – Ipiranga, São Paulo, Brazil Universidade de São Paulo São Paulo Brazil

**Keywords:** Atlántico, Bolívar, Caribbean, *
Eburodacrys
*, taxonomy, longhorn beetle, tropical dry forest

## Abstract

Two new species of *Beraba* from Colombia (Bolívar) are described: *Berabaanae***sp. n.** and *Berabaangeli***sp. n.** The most recent key to species of the genus was modified to incorporate the new species. The male of *Berabalimpida* Martins, 1997 is described and illustrated for the first time. Moreover, the geographical distribution of 12 species of Eburiini is expanded.

## Introduction

The tribe Eburiini is currently composed of 24 genera and 268 species, all of them with a geographical distribution restricted to North, Central (including the Caribbean) and South America (Botero and Monné 2018).

The genus *Beraba* Martins, 1997 was described to gather together the species described initially in *Eburia* Lacordaire, 1830 having a bright aspect, antennomere III longer than IV, and femora with spines restricted to the inner margin. Later, Galileo and Martins (2000) transferred *Eburodacryscheilaria* Martins, 1967 to the genus. Currently, the genus includes 18 species distributed from Panama to South America. Three of these species – *B.inermis* Martins & Galileo, 2002, *B.marica* Galileo & Martins, 2000 and *B.piriana* Martins, 1997 – are known to occur in Colombia (Botero 2015; Tavakilian and Chevillote 2018).

In this work two new species of *Beraba* are described, *B.anae* sp. n. and *B.angeli* sp. n.; the male of *B.limpida* Martins, 1997 is redescribed and the geographical distribution is expanded for 12 species. The key proposed by Botero (2015) for *Beraba* is modified to include the new species.

## Material and methods

The material examined was obtained in Colombia, from the tropical dry forest in the Reserva La Flecha (RLF), Bolívar and the Reserva Campesina la Montaña (RCM), Atlántico. These locations were sampled from February to May 2018 by using a UV light trap, white light trap, manual capture, and the sampling was supplemented by visits to entomological collections.

The material currently resides in the following institutions, which are subsequently referred to by their acronyms:

**ANDES-E** Colección Entomológica, Museo de Historia Natural, Universidad de Los Andes, Bogotá, Colombia (Yiselle Patricia Cano);

**IAVH** Instituto de Investigaciones de Recursos Biológicos “Alexander von Humboldt”, Villa de Leyva, Colombia (Jhon Cesar Neita);

**MPUJ** Pontificia Universidad Javeriana, Bogotá, Colombia (Igor Dimitri Forero, Giovanny Fagua);

**MZSP** Museu de Zoologia, Universidade de São Paulo, São Paulo, Brazil (Sônia Casari, Antonio Santos-Silva);

**UARC** Universidad del Atlántico, Puerto Colombia, Colombia (Neis José Martínez).

Photographs were taken with a Canon EOS Rebel T3i DSLR camera, Canon MP-E 65mm f/2.8 1–5× macro lens, controlled by Zerene Stacker focus stacking software. Measurements were taken in “mm” using a measuring ocular Hensoldt/Wetzlar - Mess 10 in the Leica MZ6 stereomicroscope, also used in the study of the specimens. References and geographical distributions were ascertained in Martínez (2000), Monné (2018) and Tavakilian and Chevillotte (2018) catalogs.

## Results

### Cerambycidae Latreille, 1802

#### Cerambycinae Latreille, 1802

##### Eburiini Blanchard, 1845

###### Beraba anae sp. n.

Taxon classificationAnimaliaColeopteraCerambycidae

http://zoobank.org/8E564587-C14F-4F52-8D96-14D8AB7CDA29

Figs 1–5

####### Type material.

Holotype female from Colombia, Bolívar: San Jacinto (Reserva La Flecha, 324 m, 09°51'12.4"N, 75°10'41.4"W, tropical dry forest), 15.IV.2018, García, K coll., UV light trap, MPUJ_ENT 0064073 (MPUJ). Paratype, male from Colombia, Bolívar: San Jacinto (Reserva La Flecha, 324 m, 09°51'12.4"N, 75°10'41.4"W, tropical dry forest), 15.IV.2018, García, K coll., white light trap (UARC).

####### Diagnosis.

Surface of pronotum smooth with pronotal tubercles of same color as remainder; eburneous callosities subrounded, posterior ones placed at same level and subequal in size; elytral costae absent; meso- and metafemora with a long spine; elytral apex truncate, with spine at outer margin.

####### Description.

***Female.*** Integument brownish orange. Apex of lateral tubercles of prothorax darker. Antennae, femora and tibiae slightly lighter. Scutellum brown. Posterior region of anterior eburneous callosity and, anterior and posterior region of posterior eburneous callosities black.

Body covered with long, erect sparse setae, denser at inner face of protibiae, protarsomeres and basal antennomeres.

**Head**. Upper ocular lobes well separated, distance between them about 4 times width of upper lobe. Antennae exceeding elytral apices at antennomere VIII. Antennal formula (ratio) based on length of antennomere III: scape = 0.73; pedicel = 0.15; IV = 0.84; V = 0.82; VI = 0.82; VII = 0.82; VIII = 0.80; IX = 0.75; X = 0.64; XI = 0.76.

**Thorax**. Prothorax (including lateral tubercles) slightly longer than wide. Sides of prothorax with tubercles distinct, acute at apex; antemedian gibbosity slightly elevated. Surface of pronotum smooth, with sparse shallow punctures, and a few sparse long setae arising from each puncture. Pronotum with two anterior tubercles weakly elevated, rounded at apex, and a centro-longitudinal slightly elevated gibbosity. Prosternum smooth with a few sparse long erect setae. Prosternal process expanded at apex, width at narrowest point equal to one fifth of procoxal cavity width. Prosternal process, meso and metaventrite covered with dense goldish pubescence, denser and longer at lateral regions. Elytra about three times longer than prothorax; surface with moderately dense, coarse punctures on anterior half, finer and shallow toward apex. Each elytron with 3 eburneous callosities: one anterior, elliptical; two posterior slightly elongated, subequal, about one fifth of elytral length, not distinctly separated, external starting slightly ahead of inner one; elytral costae absent. Apex of elytra truncate, with external spine, and a very small sutural spicule.

**Abdomen**. Ventrites finely, sparsely punctate, sparser on median region; with a few long, sparse whitish setae. Apex of ventrite V slightly emarginate.

**Variability.** In the paratype (male), the posterior eburneous callosities start at the same point. The ventrite V is shorter and square-shaped in males, with apex truncate.

**Measurements.** Holotype female: Total length, 10.6; prothorax length, 2.4; prothorax width at its widest point, 2.1; elytral length, 7.0; humeral width, 2.4. Paratype male: Total length, 11.3; prothorax length, 2.3; prothorax width at its widest point, 2.0; elytral length, 7.0; humeral width, 2.2.

####### Etymology.

The species epithet is in honor of Ana López Guerrero, mother of the first author, in appreciation of her love and support through all my life, the reason for all of my achievements.

####### Discussion.

*Berabaanae* sp. n. is similar to *B.iuba* Martins, 1997 (Figs 6–7) and *B.moema* Martins, 1997 in having only one elliptical eburneous callosity at the anterior region of each elytron, tubercles of the pronotum concolor with remaining surface and posterior eburneous callosities starting at the same level. *Berabaanae* sp. n. differs from *B.iuba* in having the posterior eburneous callosities elliptical in shape and subequal in size (in *B.iuba*, the posterior callosities are more elongated and the inner one is shorter than the external one), spined elytral apex (unarmed in *B.iuba*), and in the long meso- and metafemoral spines, longer than the scape (shorter than the scape in *B.iuba*). *Berabaanae* sp. n. differs from *B.moema* in the surface of the pronotum smooth, eburneous callosities narrowed and subrounded, posterior callosities with similar size, elytral costae absent, and elytral apex truncate. In *B.moema*, the surface of pronotum is coarsely punctuate, eburneous callosities are narrowed with the posteroexternal at least twice length of the internal, the elytral costae are visible, and the elytral apex is obliquely truncate.

**Figures 1–7.**
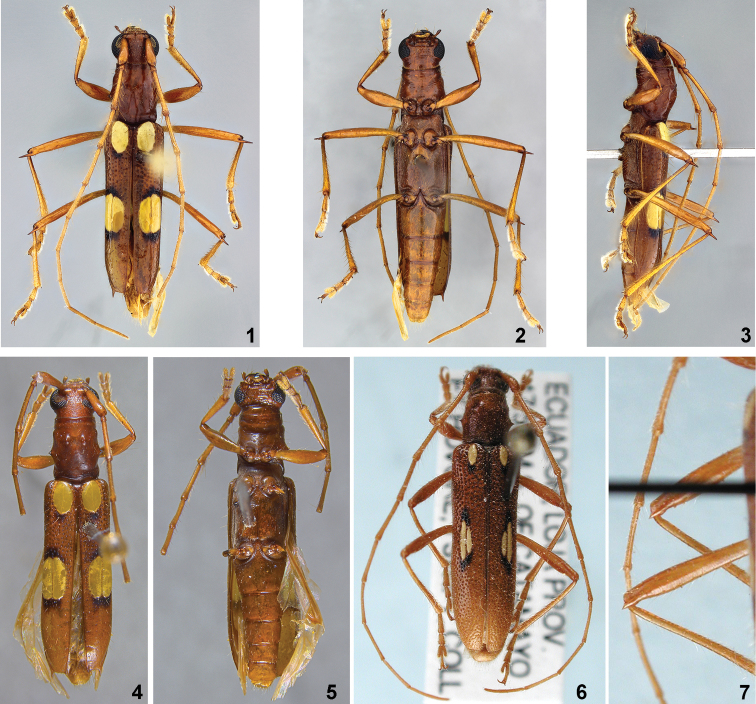
**1–5***Berabaanae* sp. nov.: **1** dorsal view, holotype female **2** ventral view, holotype female **3** lateral view, holotype female **4** dorsal view, paratype male **5** ventral view, paratype male **6–7***Berabaiuba* Martins, 1997.

###### Beraba angeli sp. n.

Taxon classificationAnimaliaColeopteraCerambycidae

http://zoobank.org/596B3878-6A51-4F68-B659-1CAB4D72B47E

Figs 8–11

####### Type material.

Holotype male from Colombia, Bolívar: San Jacinto (Reserva La Flecha, 324 m, 09°51'12.4"N, 75°10'41.4"W, tropical dry forest), 16.IV.2018, García, K. coll., white light trap, MPUJ_ENT 0064074 (MPUJ). Paratype, male from Colombia, Bolívar: San Jacinto (Reserva La Flecha, 324 m, 09°51'12.4"N, 75°10'41.4"W, tropical dry forest), 15.IV.2018, García, K. coll., UV light trap (UARC).

####### Diagnosis.

Surface of pronotum with wrinkles; pronotal tubercles black and well-elevated; males with sexual punctation on prosternum; femoral spines of same color as remainder; eburneous callosities with similar size; elytral apex with external spine.

####### Description.

***Male.*** Integument brownish orange, legs slightly lighter. Pronotal tubercles, posterior region of anterior eburneous callosity, and anterior and posterior region of posterior eburneous callosities black.

Body covered with long, erect and sparse yellowish setae.

**Head**. Upper ocular lobes well separated, distance between them about 3 times width of upper lobe. Antennae exceeding elytral apices at antennomere VIII. Antennal formula (ratio) based on length of antennomere III: scape = 0.57; pedicel = 0.11; IV = 0.83; V = 0.83; VI = 0.83; VII = 0.80; VIII = 0.74; IX = 0.69; X = 0.63; XI = 0.80.

**Thorax**. Prothorax (including lateral tubercles) longer than wide. Sides of prothorax with tubercles distinct and acute at apex. Surface of pronotum coarsely punctate with transverse wrinkles, more distinct on posterior half, with a few long sparse whitish setae. Pronotum with two anterior tubercles well elevated, rounded at apex, and a centro-longitudinal slightly elevated gibbosity. Prosternum with transverse sulcus, glabrous, with long yellowish setae, finely transversely striate, coarsely punctate on posterior half, with two well-defined areas of sexual punctation. Prosternal process expanded at apex, width at narrowest point equal to one fourth of procoxal cavity width. Prosternal process, meso- and metaventrite covered with dense golden pubescence, denser and longer laterally. Elytra about three times longer than prothorax; surface with moderately dense, coarse punctures on anterior half, finer and shallower toward apex. Each elytron with 3 subrounded eburneous callosities: one anterior; two posterior subfused, subequal, starting at same level, external slightly curved. Elytral costae absent. Apex of elytra truncate, with external spine and dentiform projection at sutural angle.

**Abdomen**. Ventrites finely sparsely punctate, sparser on median region; with a few moderately long, sparse yellowish setae. Apex of ventrite V truncate.

**Measurements.** Holotype male: total length, 9.5; prothorax length, 1.9; prothorax width at its widest point, 1.7; elytral length, 6.3; humeral width, 2.0. Paratype male: total length, 11.9; prothorax length, 2.5; prothorax width at its widest point, 2.5; elytral length, 8.1; humeral width: 2.7.

####### Etymology.

The species epithet is in honor of Angel García Hernandez, father of the first author, as a thank you for all the support, love and happiness he has given to me.

####### Discussion.

*Berabaangeli* sp. n. is similar to *B.marica* Galileo & Martins, 1999 (Figs 12–14) and *B.inermis* Martins & Galileo, 2002 in having only one elliptical eburneous callosity on the anterior region of each elytron, tubercles of the pronotum black, and surface of the pronotum only with wrinkles or with wrinkles and some interspersed punctures. *Berabaangeli* sp. n. differs of *B.marica* in having the pronotal tubercles distinctly elevated, prosternum with areas of sexual punctation (Fig. 11), and the male ventrites not modified (in *B.marica* the tubercles are slightly elevated, prosternum does not show sexual punctation, and ventrites II–IV show depressed areas with long yellowish setae on the posterior margin in males, as in Fig. 14). *Berabaangeli* sp. n. differs of *B.inermis* in the posterior eburneous callosities of similar size, and apex of the elytra with an external spine (in *B.inermis*, the posteroexternal eburneous callosities is, at least, twice the length of internal, and the external apex of the elytra is unarmed).

**Figures 8–14.**
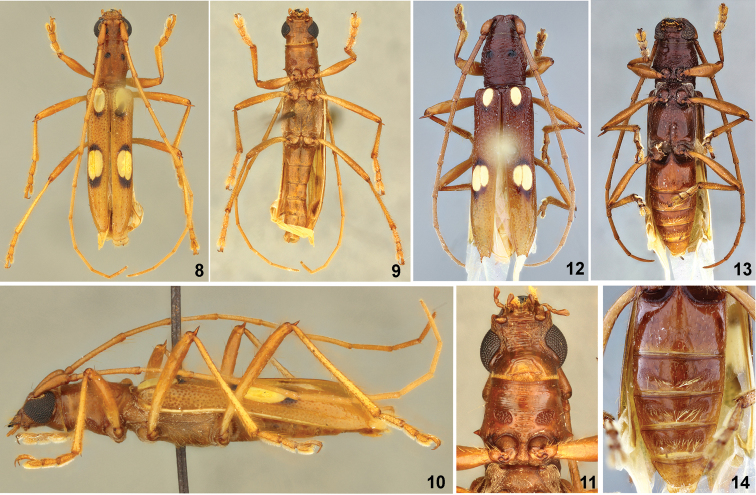
**8–11***Berabaangeli* sp. nov., holotype, male: **8** dorsal view **9** ventral view **10** lateral view **11** detail of prosternum. **12–14***Berabamarica* Martins & Galileo, 1999: **12** dorsal view **13** ventral view **14** detail of ventrites.

According to the most recent key to species of *Beraba* (Botero, 2015), the two new species can be inserted as follows:

**Table:** 

7(4)	Tubercles of pronotum of same color as remainder of pronotum	**8**
–	Tubercles of pronotum black (contrasting in color from remainder of pronotum)	**11**
8(7)	Posteroexternal eburneous callosity of elytra placed at beginning of apical third and distant internal callosity. Brazil (Rio de Janeiro)	***B.angusticollis* (Zajciw, 1961)**
–	Posterior eburneous callosities of elytra placed at same level	**9**
9(8)	Meso- and metafemora with short inner spine, shorter than length of scape; elytral apex unarmed (Figs 6–7). Colombia (Bolívar), Ecuador (Pichincha)	***B.iuba* Martins, 1997**
–	Meso- and metafemora with long inner spine, longer than length of scape; elytral apex with spines	**10**
10(9)	Surface of pronotum coarsely punctate; eburneous callosities narrowed, posteroexternal eburneous callosity at least twice length of internal; elytral costae visible; elytral apex obliquely truncate. Ecuador (El Oro, Guayas, Manabi)	***B.moema* Martins, 1997**
–	Surface of pronotum smooth; eburneous callosities wider, subrounded; posterior eburneous callosities with similar size; elytral costae absent; elytral apex truncate (Figs 1–5). Colombia (Bolívar)	***B.anae* sp. n.**
11(7)	Apex and spines of femora of same color as remainder	**12**
–	Apex and spines of femora black, contrasting with adjacent color	**16**
12(11)	Surface of pronotum only with wrinkles or with wrinkles and some interspersed punctures	**13**
–	Surface of pronotum only with punctures, without wrinkles	**15**
13(12)	Posteroexternal eburneous callosities at least twice length of internal; external apex of elytra unarmed. Colombia (Amazonas, Bolívar, Cundinamarca, Valle del Cauca)	***B.inermis* Martins & Galileo, 2002**
–	Posterior eburneous callosities with similar size; apex of elytra with external spine	**14**
14(13)	Pronotal tubercles slightly elevated; prosternum without sexual punctation; ventrites II-IV of males with depressed areas and with long yellowish setae on posterior margin of those areas (Figs 12–14). Colombia (Atlántico, Bolívar, Magdalena, Santander)	***B.marica* Galileo & Martins, 1999**
–	Pronotal tubercles distinctly elevated; prosternum with areas of sexual punctation (Fig. 11); ventrites of males not modified (Figs 8–11). Colombia (Bolívar)	***B.angeli* sp. n.**
15(12)	Basal eburneous callosities narrowed and elongated; elytral costae visible behind posterior callosities. Bolivia (Santa Cruz)	***B.tate* Galileo & Martins, 2010**
–	Basal eburneous callosities short and subrounded; without elytral costae visible behind posterior callosities (Figs 15–19). Colombia (Bolívar), Venezuela	***B.limpida* Martins, 1997**
16(11)	Pronotum rugosely punctate	**17**
–	Pronotum smooth or only with microsculpture	**19**
17(16)	Scape black or darker than flagellomeres; prothorax subparallel-sided; eburneous callosities elongate and thin. Brazil (Goiás, Maranhão, Mato Grosso, Piauí)	***B.decora* (Zajciw, 1961)**
–	Scape with same color as flagellomeres; prothorax curved at sides or narrowed toward anterior margin; eburneous callosities elliptical	**18**
18(17)	Lateral tubercle of prothorax small; posterior eburneous callosities starting anteriorly at same level; apex of elytra with black area. French Guiana	***B.odettae* Martins & Galileo, 2008**
–	Lateral tubercle of prothorax long and acute; posteroexternal eburneous callosity starting behind internal one; apex of elytra without black area. Peru	***B.spinosa* (Zajciw, 1967)**
19(16)	Prothorax longer than wide, anterior region of epipleura without projection, metafemora exceeding elytral apex. Brazil (Amazonas), French Guiana	***B.cauera* Galileo & Martins, 1999**
–	Prothorax as long as wide; anterior region of epipleura with projection, metafemora not exceeding elytral apex. Brazil (Mato Grosso do Sul), Bolivia (Cochabamba, Santa Cruz), Paraguay	***B.cheilaria* (Martins, 1967)**

###### Beraba limpida

Taxon classificationAnimaliaColeopteraCerambycidae

Martins, 1997

Figs 15–19

####### Material examined.

Colombia, Bolívar: San Jacinto (Reserva La Flecha, 09°51'12.4"N, 75°10'41.4"W, tropical dry forest), 1 male, 27.IV.2017, I. Mendoza coll., light trap (UARC). Venezuela, Aragua: El Limón, 1 male, 23.V.1997, F. Fernandez coll., mercury light (MZSP).

####### Redescription.

**Male.** Integument dorsally orange, brownish orange ventrally. Posterior region of head, pronotum, lateral tubercles of prothorax and legs brownish orange. Elytra and scutellum yellowish orange. Pronotal tubercles, posterior region of anterior eburneous callosities and, region around posterior eburneous callosities black.

Body covered with long, erect, sparse setae, denser on inner surface of tibiae, tarsomeres and basal antennomeres.

**Head**. Posterior region of head, scape and basal antennomeres with dense punctuation. Distance between upper lobes about three times width of upper lobe. Antennae exceeding elytral apices at apex of antennomere VIII. Prothorax (including lateral tubercles) 1.14 times longer than wide; lateral tubercles distinctly visible, acute at apex.

**Thorax**. Surface of pronotum with coarse dense punctuation; with two anterior elevated tubercles rounded at apex. Prosternum smooth on central region, with long, erect setae and a few punctures, with evident sexual punctation at lateral sides concentrated on subrounded areas (Figs 16, 18). Coxae and mesoventral process covered with dense whitish pubescence. Meso- and metaventrite with long erect setae and covered with dense whitish pubescence laterally. Femora and tibiae fine and long; apex of meso- and meta-femora with long inner spine.

**Figures 15–19.**
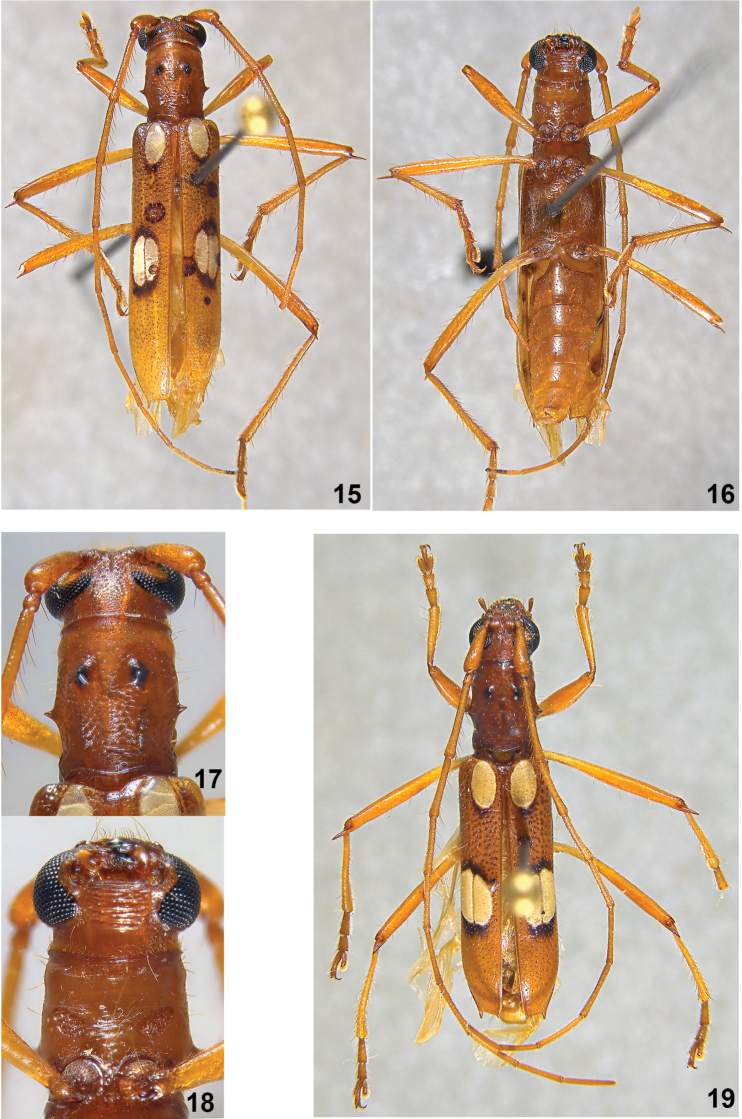
*Berabalimpida* Martins, 1997. **15–18** male: **15** dorsal view **16** ventral view **17** detail of pronotum **18** detail of prosternum **19** female, dorsal view.

Elytra about 3.5 times longer than prothorax; surface with dense, coarse punctures basally, finer and shallow toward apex. Each elytron with three eburneous callosities: one basal, elliptical; two posterior, slightly elongated, inner one slightly smaller than external one, not distinctly separated from each other. Posteroexternal callosity about one fifth of elytral length, starting ahead of internal one. Elytral costae absent. Apex of elytra with external long spine, about 0.8 times as long as the pedicel and with acute sutural projection.

**Measurements**. Male. Total length, 10.3; prothorax length, 2.1; prothorax width at its widest point, 1.9; elytral length, 7.3; humeral width, 2.0.

####### Discussion.

*Berabalimpida* was described by Martins (1997) based on a single female specimen, and until now the male remained unknown. Among the known males of *Beraba*, just one species has sexual punctation, *B.piriana* Martins, 1997. The sexual punctation in this species covers the entire prosternum and extends to the lateral region of the pronotum. Herein, we report sexual punctation for the first time in other two species of *Beraba*: *B.limpida* and *B.angeli* sp. n. In those species, the sexual punctation covers the entire surface of the pronotum, and is concentrated in subrounded areas on sides of the prosternum (Figs 9, 11, 16, 18).

One specimen of *Berabatate* Galileo & Martins, 2010 was illustrated by Galileo et al. (2008) as being *B.limpida*. Later, Galileo and Martins (2010) recognized that this specimen belongs to a new species, and described it as *B.tate*. However, Galileo et al. (2008) remains wrongly listed in the references on *B.limpida* (see Monné 2018; Tavakilian and Chevillotte 2018). In order to correct this error, we point out that this reference should appear on *B.tate*.

##### New geographical records

###### Beraba inermis

Taxon classificationAnimaliaColeopteraCerambycidae

Martins & Galileo, 2002

####### Geographical distribution.

Colombia (Cundinamarca, Valle del Cauca). New department records are added: Amazonas and Bolívar (Colombia).

####### Specimens examined.

Colombia, Amazonas: Leticia, 1 female, 1.V.2001, Sarmiento Paula coll., Andes-E1162 (ANDES-E); Bolívar: San Jacinto (Reserva La Flecha, 09°51'12.4"N, 75°10'41.4"W, tropical dry forest), 1 male, 27.IV.2017, I. Mendoza coll., light trap (UARC).

###### Beraba iuba

Taxon classificationAnimaliaColeopteraCerambycidae

Martins, 1997

####### Geographical distribution.

Ecuador. A new country record from Colombia (Bolívar) is added.

####### Specimen examined.

Colombia, Bolívar: San Jacinto (Reserva La Flecha, 09°51'12.4"N, 75°10'41.4"W, tropical dry forest), 1 male, 27.IV.2017, I. Mendoza coll., light trap (UARC).

###### Beraba limpida

Taxon classificationAnimaliaColeopteraCerambycidae

Martins, 1997

####### Geographical distribution.

Venezuela. A new country record from Colombia (Bolívar) is added.

####### Specimens examined.

Colombia, Bolívar: San Jacinto (Reserva La Flecha, 09°51'12.4"N, 75°10'41.4"W, tropical dry forest), 1 male, 1 female, 27.IV.2017, I. Mendoza coll., light trap (UARC).

###### Beraba marica

Taxon classificationAnimaliaColeopteraCerambycidae

Galileo & Martins, 2000

####### Geographical distribution.

Colombia (Bolívar). New department records are added: Atlántico, Magdalena and Santander (Colombia).

####### Specimen examined.

Colombia, Atlántico: Usiacurí; (Reserva Campesina La Montaña, 10°46'2.6"N, 75°0.2'34"W, tropical dry forest), 1 male, 14.V.2018, K. García coll., UV light trap (UARC); Magdalena: (Road Minka-Cerro Kennedy, 11°07'31"N, 74°06'07"W, 1000 m), 1 female, 7–8.VI.2018, V. Sinyaev coll. (MZSP); Santander: Carmen de Churucí (Vereda La Belleza, Finca Santiago, Campamento, 06°34'49.5"N, 73°34'15.1"W, 801 m, Bosque húmedo tropical), 18–25.II.2018, Neita, J. C. coll., “captura manual”, CAS–00039 (IAVH).

###### Beraba piriana

Taxon classificationAnimaliaColeopteraCerambycidae

Martins, 1997

####### Geographical distribution.

Panama, Colombia (Magdalena). New department records are added: Atlántico and Bolívar (Colombia).

####### Specimens examined.

Colombia, Atlántico: Baranoa (10°47'56.04"N, 74°55'19.56"W), 2 males, 4.IV.2017, I. Mendoza coll., manual capture (UARC); Usiacurí; (Reserva Campesina La Montaña, 10°46'2.6"N, 75°0.2'34"W, tropical dry forest), 1 male, 1 female, 12.V.2018, K. García coll., white light trap (UARC); 1 female, 12.V.2018, K. García coll., UV light trap (MZSP); 1 female, 12.V.2018, K. García coll., manual capture (UARC); Bolívar: San Jacinto (Reserva La Flecha, 09°51'12.4"N, 75°10'41.4"W, tropical dry forest), 2 males, 1 female, 16.IV.2018, K. García col., UV light trap (UARC); Bolívar: San Jacinto (Reserva La Flecha, 09°51'12.4"N, 75°10'41.4"W, tropical dry forest), 1 male, 27.IV.2017, I. Mendoza coll., light trap (UARC); Bolívar: San Jacinto (Reserva La Flecha, 09°51'12.4"N, 75°10'41.4"W, tropical dry forest), 1 female, 6.VI.2016, J. Barraza coll., van someren-rydon (UARC).

###### Eburodacrys asperula

Taxon classificationAnimaliaColeopteraCerambycidae

Bates, 1880

####### Geographical distribution.

Honduras, Mexico (Veracruz), Guatemala, Costa Rica, Panama, Venezuela. A new country record from Colombia (Magdalena) is added.

####### Specimens examined.

Colombia, Magdalena (Road Minka-Cerro Kennedy, 11°07'31"N, 74°06'07"W, 1000 m), 1 male, 1 female, 7–8.VI.2018, V. Sinyaev coll. (MZSP).

###### Eburodacrys callixantha

Taxon classificationAnimaliaColeopteraCerambycidae

Bates, 1872

####### Geographical distribution.

Honduras, Mexico (Jalisco, Oaxaca), Nicaragua, Panama, Venezuela. A new country record from Colombia (Magdalena) is added.

####### Specimen examined.

Colombia, Magdalena: San Pablo; (La Clarita, 10°52'37.3"N, 74°08'03.9"W), 3 males, 28.V.2017, I. Mendoza and L. Martinez coll., light trap (UARC).

###### Eburodacrys coalescens

Taxon classificationAnimaliaColeopteraCerambycidae

Bates, 1884

####### Geographical distribution.

Mexico, Guatemala, Honduras, Nicaragua, Costa Rica, Panama. A new country record from Colombia (Atlántico) is added.

####### Specimens examined.

Colombia, Atlántico: Usiacurí; (Reserva Campesina La Montaña, 10°46'2.6"N, 75°0.2'34"W, tropical dry forest), 2 females, 12.V.2018, K. García coll., white light trap (MZSP, UARC).

###### Eburodacrys havanensis

Taxon classificationAnimaliaColeopteraCerambycidae

Chevrolat, 1862

####### Geographical distribution.

Cuba, Mexico, Nicaragua, Costa Rica, Panama, Colombia (Antioquia, Cundinamarca, Huila, Meta, Putumayo, Quindío, Santander, Tolima), Venezuela, Bolivia (Beni, Santa Cruz), Brazil (Mato Grosso, Mato Grosso do Sul, Goiás, Distrito Federal, Maranhão, Piauí, Pernambuco, Bahia, Minas Gerais, Espírito Santo, Rio de Janeiro, São Paulo, Paraná, Santa Catarina, Rio Grande do Sul), Paraguay. A new department record from Bolívar (Colombia) is added.

####### Specimens examined.

Colombia, Bolívar: San Jacinto (Reserva La Flecha, 09°51'12.4"N, 75°10'41.4"W, tropical dry forest), 1 male, 13.IV.2018, K. García coll., white light trap (UARC); 1 male, 27.IV.2017, I. Mendoza coll., light trap (UARC).

###### Eburodacrys moruna

Taxon classificationAnimaliaColeopteraCerambycidae

Martins, 1997

####### Geographical distribution.

Colombia (Magdalena). New department records are added: Atlántico and Bolívar (Colombia).

####### Specimens examined.

Colombia, Atlántico: Usiacurí (Reserva Campesina la Montaña, 10°46'2.6"N, 75°0.2'34"W, tropical dry forest), 1 female, 14.VI.2018, J. Sarmiento coll., pitfall (UARC). Bolívar: San Jacinto (Reserva La Flecha, 09°51'12.4"N, 75°10'41.4"W, tropical dry forest), 1 female, 27.IV.2017, I. Mendoza coll., light trap (UARC).

###### Eburodacrys santossilvai

Taxon classificationAnimaliaColeopteraCerambycidae

Botero, 2017

####### Geographical distribution.

Venezuela. A new country record from Colombia (Atlántico, Bolívar) is added.

####### Specimens examined.

Colombia, Atlántico: Usiacurí; (Reserva Campesina La Montaña, 10°46'2.6"N, 75°0.2'34"O, tropical dry forest), 1 female, 14.V.2018, K. García coll., UV light trap (UARC); Bolívar: San Jacinto (Reserva La Flecha, 09°51'12.4"N, 75°10'41.4"W, tropical dry forest), 1 female, 16.III.2018, K. García coll., UV light trap (UARC); 2 males, 27.IV.2017, I. Mendoza coll., light trap (UARC).

###### Eburodacrys triocellata

Taxon classificationAnimaliaColeopteraCerambycidae

(Stal, 1857)

####### Geographical distribution.

Mexico, Guatemala, Nicaragua, Costa Rica, Panama, Colombia (Antioquia, Arauca, Boyacá, Caldas, Cesar, Cundinamarca, Magdalena, Meta, Tolima, Valle del Cauca), Venezuela. New department records are added: Amazonas, Atlántico and Bolívar (Colombia).

####### Specimen examined.

Colombia, Amazonas: Leticia, female, 1.V.2001, Daniel Matute coll., Andes-E384 (ANDES-E); Atlántico: Usiacurí; (Reserva Campesina La Montaña, 10°46'2.6"N, 75°0.2'34"W, tropical dry forest), 1 female, 15.II.2018, K. García coll., white light trap (UARC); 1 female, 12.V.2018, K. García coll., manual capture (UARC); 2 females, 12.V.2018, K. García coll., white light trap (UARC); Bolívar: San Jacinto (Reserva La Flecha, 09°51'12.4"N, 75°10'41.4"W, tropical dry forest), 1 male, 1 female, 16.IV.2018, K. García coll., UV light trap (UARC); 2 male, 5 females, 27.IV.2017, I. Mendoza coll., light trap (UARC).

## Supplementary Material

XML Treatment for Beraba anae

XML Treatment for Beraba angeli

XML Treatment for Beraba limpida

XML Treatment for Beraba inermis

XML Treatment for Beraba iuba

XML Treatment for Beraba limpida

XML Treatment for Beraba marica

XML Treatment for Beraba piriana

XML Treatment for Eburodacrys asperula

XML Treatment for Eburodacrys callixantha

XML Treatment for Eburodacrys coalescens

XML Treatment for Eburodacrys havanensis

XML Treatment for Eburodacrys moruna

XML Treatment for Eburodacrys santossilvai

XML Treatment for Eburodacrys triocellata
